# Burosumab improves alkaline phosphatase and rickets severity in X-linked hypophosphatemia: 3-year SUNFLOWER analysis

**DOI:** 10.1016/j.bonr.2026.101944

**Published:** 2026-08-30

**Authors:** Keiichi Ozono, Hee Gyung Kang, Noriyuki Namba, Nobuaki Ito, Takuo Kubota, Ayumi Shintani, Ryota Kawai, Osamu Miyazaki, Masanori Kanematsu, Haruka Ishii, Toshimi Michigami

**Affiliations:** a Center for Promoting Treatment of Intractable Diseases, ISEIKAI International General Hospital, Osaka, Japan; b Department of Pediatric Nephrology, Seoul National University Children's Hospital, Seoul, Republic of Korea; c Division of Pediatrics and Perinatology, Faculty of Medicine, Tottori University, Tottori, Japan; d Division of Therapeutic Development for Intractable Bone Diseases, Graduate School of Medicine and Faculty of Medicine, The University of Tokyo, Tokyo, Japan; e Department of Pediatric Nephrology and Metabolism, Osaka Women's and Children's Hospital, Osaka Prefectural Hospital Organization, Osaka, Japan; f Department of Medical Statistics, Graduate School of Medicine, Osaka Metropolitan University, Osaka, Japan; g Department of Radiology, National Center for Child Health and Development, Tokyo, Japan; h Medical Affairs Department, Kyowa Kirin Co., Ltd., Tokyo, Japan; i Department of Bone and Mineral Research, Osaka Women's and Children's Hospital, Osaka Prefectural Hospital Organization, Osaka, Japan

**Keywords:** Osteomalacia, Rickets, Phosphate metabolism, Bone metabolism marker, Patient-reported outcomes, Quality of life

## Abstract

SUNFLOWER is a 10-year longitudinal observational cohort study of patients with X-linked hypophosphatemia (XLH) in Japan and South Korea. This report describes the 3-year cut-off of SUNFLOWER, including data from 1 year of burosumab administration (burosumab-treated group) and 1 year after providing informed consent (non-burosumab-treated group). The real-world effectiveness of burosumab and the associations between bone metabolism markers and quality of life (QoL)/patient-reported outcomes (PROs) and rickets severity scores (RSS)/height over 1 year in burosumab-treated patients with XLH were assessed. As burosumab had not been launched in South Korea at the time of cut-off, data from South Korea were not available. The main outcome measures were changes in serum parameters, and correlations between alkaline phosphatase (ALP) levels and RSS and height (children), and between ALP (children) or bone-specific ALP (BALP; adults) levels and QoL/PROs and motor functions. In total, 146 patients were enrolled, including 69 (47.3%) children and 77 (52.7%) adults. After 1 year of treatment, burosumab-treated patients (43 [62.3%] children, 29 [37.7%] adults) had improved serum phosphate levels, 1,25-dihydroxyvitamin D, and ALP/BALP compared with pre-treatment levels. In children, RSS changes significantly correlated with ALP changes (correlation coefficient: 0.806, *p* < 0.001), but height changes did not show significant correlation. There were no significant correlations between changes in ALP/BALP and QoL/PRO assessments or motor function. In conclusion, decreased ALP correlated with an improvement in rickets severity score in Japanese children with XLH, suggesting that ALP may be a useful marker for monitoring both the severity and improvement of rickets/osteomalacia.

**Clinical trial registration:**

NCT03745521 and UMIN000031605.

## Highlights

• Real-world effectiveness of burosumab in patients with X-linked hypophosphatemia.
• Out of 146 patients, 43 adults and 29 children were treated with burosumab.
• At 1 year, burosumab improved phosphate, 1,25-dihydroxyvitamin D, and ALP/BALP.
• In children, the changes in rickets severity scores correlated with ALP changes.
• ALP may be a marker for severity/improvement of rickets/osteomalacia.

## 1 Introduction

X-linked hypophosphatemia (XLH) is a rare genetic disorder caused by pathogenic variants in the phosphate-regulating gene with homologies to endopeptidases on the X chromosome (*PHEX*) (Carpenter et al., 2011; Baroncelli and Mora, 2021; Linglart et al., 2014; Lambert et al., 2019). These variants cause excess fibroblast growth factor 23 (FGF23), reduced phosphate reabsorption in the kidneys, and suppression of phosphate absorption from the intestine by decreased 1,25-dihydroxyvitamin D [1,25(OH)_2_D] levels (Carpenter et al., 2011; Baroncelli and Mora, 2021; Linglart et al., 2014; Lambert et al., 2019). This results in chronic hypophosphatemia and contributes to the varied symptoms of XLH. XLH can present as rickets, dental problems, abnormal gait, and short stature in childhood, and bone pain, fatigue, and an increased risk of fractures and pseudofractures in adulthood (Carpenter et al., 2011; Baroncelli and Mora, 2021; Linglart et al., 2014; Lambert et al., 2019).

Oral phosphate and active vitamin D (Pi/D) are used to treat XLH by reversing low phosphate levels (Haffner et al., 2019). However, this treatment has a burdensome dosing regimen and adherence issues, does not address the elevated levels of FGF23, and normalization of serum phosphate is generally difficult. Moreover, Pi/D can result in renal complications (such as nephrocalcinosis) and secondary/tertiary hyperparathyroidism (Harada et al., 2021).

Burosumab is a fully human monoclonal antibody that binds to FGF23, reversing reduction in phosphate reabsorption and increasing serum phosphate levels (Imel, 2021). Clinical trials and observational studies have shown that burosumab treatment effectively improves serum phosphate levels, rickets severity, height, healing of fractures/pseudofractures, motor function, and quality of life (QoL) in adults and children (Insogna et al., 2019; Whyte et al., 2019; Carpenter et al., 2018; Paloian et al., 2022; Ward et al., 2024; Liu et al., 2025; Florenzano et al., 2025; Ward et al., 2026). However, few studies have assessed the real-world effectiveness and safety of burosumab treatment prospectively in patients with XLH (Paloian et al., 2022; Florenzano et al., 2025; Ward et al., 2026). Additionally, symptom improvement can occur in some patients who do not reach the normal phosphate range (Walker et al., 2023), whereas other patients may have improved serum phosphate levels without physical improvements, suggesting that serum phosphate alone is insufficient for evaluating the effect of treatment (Walker et al., 2023; Seefried et al., 2021; Ito et al., 2022; Skrinar et al., 2019). There are limited reports of factors associated with clinical endpoint improvements (Seefried et al., 2021; Ito et al., 2022; Skrinar et al., 2019). While some studies have evaluated the correlation between phosphate and QoL (Che et al., 2016), to our knowledge, no previous study has investigated the correlation between bone metabolism markers and QoL/patient-reported outcomes (PROs) and rickets severity score (RSS)/height in patients with XLH.

The Study of longitUdinal observatioN For patients with X-Linked hypOphosphatemic rickets/osteomalacia in collaboration With Asian partnERs (SUNFLOWER) study is a 10-year longitudinal observational cohort study of patients with XLH that is being conducted in Japan and South Korea (Kubota et al., 2020). The current report describes the 3-year cut-off of the SUNFLOWER study, including data from the first year of burosumab administration. The objectives of this analysis were to assess the real-world treatment of XLH and the effectiveness and safety of burosumab treatment. The primary objective was to evaluate correlations between bone metabolism markers and rickets severity, height, QoL/PROs, and motor function after burosumab administration. The secondary objective was to describe the effect of 1 year of treatment in patients with XLH.

## 2 Materials and methods

### 2.1 Study design

The SUNFLOWER study is a 10-year observational study being conducted at 21 centers in Japan and South Korea; the full design of the SUNFLOWER study has been reported previously (Kubota et al., 2020). This study compares outcomes in children (<18 years) and adults (≥18 years) with XLH who are receiving burosumab and those who are not. The study duration is from the enrollment date, which was between 1 April 2018 and 30 April 2022, until the end date of 30 April 2028. In this 3-year cut-off analysis, data from patients who did or did not receive burosumab treatment for up to 1 year are compared.

In this study, visits for assessments vary depending on the patient's age, with visits occurring at enrollment and 1, 2, 3, 4, 5, 7, and 10 years (end of observation) for children, and at enrollment and 1, 3, 5, 7, and 10 years (end of observation) for adults. For children who turned 18 during the study, the first visit after the patient turned 18 is considered the enrollment visit for inclusion as an adult. For patients who received burosumab treatment, assessments were performed before the start of treatment (baseline), at 3 months, 6 months, 1 year, and thereafter according to the patient's age as described above.

The study is being conducted in accordance with the Declaration of Helsinki and all relevant regulations in Japan and South Korea. Written informed consent was obtained from patients ≥18 years of age and from the parents/guardians of patients aged <16 years. For patients aged 16–17 years of age and for patients who turned 18 during the study, written informed consent was obtained from both the patients and their parents/guardian. The SUNFLOWER study was registered on ClinicalTrials.gov under the identifier NCT03745521 and on the University hospital Medical Information Network Center registry under the identifier UMIN000031605.

### 2.2 Burosumab treatment

In this study, patients with XLH are treated at their physician's discretion and in accordance with the available treatments in their country. The initial burosumab dose was determined from the patient's body weight and increased (for children only) or reduced thereafter according to phosphate levels and symptoms. No blinding methods are used.

### 2.3 Patients

As burosumab was not yet available in South Korea at the time of data cut-off (31 December 2021), this analysis includes patient data only from 18 centers in Japan. The full eligibility and exclusion criteria have been published previously (Kubota et al., 2020). Briefly, the inclusion criteria were diagnosis of XLH with a documented *PHEX* mutation, a first-degree relative with a documented *PHEX* mutation, and/or a confirmed FGF23 level > 30 pg/mL, as defined in a previous study (Endo et al., 2008); and current/previous physical examination findings or laboratory and radiographical findings of rickets or osteomalacia. Patients participating in another clinical study, or who were considered by the investigator to be inappropriate for participation in the study, were excluded.

### 2.4 Outcomes

Sex, age, height, and weight *Z*-scores, *PHEX* mutation, intact FGF23 levels, and serum parameters were evaluated at baseline.

The primary objective was to evaluate correlations between bone biomarker and clinical outcomes in patients treated with burosumab. The secondary objective was to evaluate the effect of 1 year of burosumab treatment for XLH.

In children, the endpoints for the primary objective were the correlation between alkaline phosphatase (ALP) level changes and rickets severity and height changes, and the correlation between ALP level changes and QoL/PROs and motor function changes. ALP levels were evaluated at baseline and at 3 months, 6 months, and 1 year for children treated with burosumab, and at baseline and 1 year for children not treated with burosumab. Motor function was assessed using the 6-min walk test (Anderson et al., 1996) at baseline, 6 months, and 1 year. QoL/PRO assessments were conducted at baseline, 6 months, and 1 year, and included the 10-item Short Form Health Survey for Children (Saris-Baglama et al., 2006), the EQ-5D-5L or EQ-5D-Y-3 L (EuroQol Research Foundation, 2024), the child version of the EQ visual analogue scale (EuroQol Research Foundation, 2024), the revised Faces Pain Scale (Hicks et al., 2001), the total days the child was unable to attend school because of XLH symptoms/complications or management of XLH, and the Patient-Reported Outcomes Measurement Information System (PROMIS) proxy T-score for mobility, fatigue, and pain. The RSS (Thacher et al., 2000; Thacher et al., 2019) and the Radiographical Global Impression of Change in Children (Whyte et al., 2018) were evaluated by four and three trained central evaluation committee members, respectively, and the average value was used.

In adults, the endpoint for the primary objective was the correlation between bone-specific ALP (BALP) level changes and QoL/PROs and motor function changes. BALP levels were evaluated at baseline and at 3 months, 6 months, and 1 year for adults treated with burosumab, and at baseline and 1 year for adults not treated with burosumab. Motor function was assessed using the Timed Up & Go test and grip strength (combined average of both left- and right-hand grip) at baseline, 6 months, and 1 year. QoL/PRO assessments were conducted at baseline, 6 months, and 1 year, and included the EQ-5D-5L (EuroQol Research Foundation, 2019), the EQ visual analogue scale (EuroQol Research Foundation, 2019), the Brief Pain Inventory scores (Daut et al., 1983), the Western Ontario and McMaster Universities Osteoarthritis Index score for pain (WOMAC) (Bellamy et al., 1988; Bellamy, 2021), the total days the adult was unable to attend school/work because of XLH symptoms/complications or management of XLH, and the PROMIS standardized T-score for physical function.

In both children and adults, blood and urine samples were taken at least 4 h after a meal or administration of phosphate in principle. Serum phosphate, calcium, 1,25(OH)_2_D levels, 25-hydroxyvitamin D (25[OH]D) levels, and intact parathyroid hormone levels were evaluated at baseline and at 3 months, 6 months, and 1 year for patients treated with burosumab, and at baseline and 1 year for patients not treated with burosumab.

The endpoints for the secondary objective were burosumab dosage, proportion of patients in the target range for phosphate levels at baseline and 1 year, proportion of patients in the target range for ALP/BALP levels at baseline and 1 year, height *Z*-scores, and renal calcification grade. For the target range in children, we referred to the normal range stratified by age and sex in the CALIPER reference data (Colantonio et al., 2012). The target ranges for phosphate and ALP levels by age range are shown in **Table S1**. Height Z-scores and renal calcification grades (grade 0 = normal, grade 1 = mild echogenicity visible around renal pyramids, grade 2 = severe echogenicity around renal pyramid borders and minor echogenicity of entire renal pyramids, grade 3 = severe echogenicity of entire renal pyramids, grade 4 = echogenicity indicating solitary calcified lesion at the tip of renal pyramids) were assessed at baseline and at 3 months, 6 months, and 1 year for patients treated with burosumab, and at baseline and 1 year for patients not treated with burosumab.

### 2.5 Statistical analyses

Categorical data were summarized as frequencies and proportions, and continuous data were summarized as number of observations, means, SDs, medians, interquartile ranges (IQRs), and ranges. Background characteristics between the groups were compared using Pearson's χ^2^ test or Fisher's exact test for categorical data and with a Mann–Whitney *U* test for continuous data.

To assess the association between ALP/BALP levels and QoL/PROs and motor function, rank correlation coefficients and their 95% confidence intervals (CIs) were calculated based on the standardized rank of changes from baseline. The change in ALP was evaluated for rank correlation by absolute value change. These rank correlation coefficients were estimated using a linear regression model that accounted for repeated measurements using a generalized estimating equation approach. In this model, the standardized rank of the change in QoL/PROs was the dependent variable and the standardized rank of the change in ALP/BALP was the independent variable. The number of days since the first prescription of burosumab was also included as an independent variable as an adjustment factor. The significance level for statistical testing was two-sided 5% (unless otherwise specified), and all statistical analyses were conducted using R version 4.3.1 (R Core Team, 2018). No statistical tests were conducted to compare changes in serum parameters over time; the proportions of patients in the target range for phosphate, ALP, and BALP before and after burosumab treatment; changes from baseline in height *Z*-scores; and renal calcification grade before and after burosumab treatment.

To evaluate the effect of 1 year of treatment in patients with XLH, patients were included in the non-burosumab group until they initiated burosumab, minimizing selection bias. Improvements in patients who received either burosumab or non-burosumab treatment for a full year were also assessed to evaluate the treatment effect in those who were able to continue either treatment.

## 3 Results

### 3.1 Patient baseline characteristics

A total of 146 patients were enrolled, including 69 (47.3%) children and 77 (52.7%) adults. Of these, 43 (62.3%) of the children received burosumab and 29 (37.7%) of the adults received burosumab. There were no significant differences between the burosumab-treated and not-treated groups in the proportions of male/female patients or patients with *PHEX* mutations, or in the median age, serum calcium, or serum intact parathyroid hormone (iPTH) levels, in either the children or adults (Table 1). Children treated with burosumab had lower median (IQR) height *Z*-scores (−2.07 [−2.69, −1.60]) than non-burosumab-treated children (−1.51 [−2.32, −0.91], *p* = 0.044). Adults who were treated with burosumab had significantly higher median (IQR) weight Z-scores and lower phosphate levels than those who did not receive burosumab (0.11 [−1.00, 0.56] vs −0.86 [−1.36, −0.08], *p* = 0.017, and 2.10 [1.90, 2.30] mg/dL vs 2.40 [2.10, 2.70] mg/dL, *p* = 0.043, respectively). No such difference was observed in children. Regarding past prescription history, most of the children in the burosumab-treated and not-treated groups received both oral phosphate and active vitamin D (97.7% vs 96.2%), while more adult patients in the burosumab-treated group received both oral phosphate and active vitamin D than those in the not-treated group (86.2% vs 64.6%) (Table 1).

**Table 1:** Patient baseline characteristics.

	Children (<18 years)				Adults (≥18 years)			
	Total (*N* = 69)	Burosumab-treated (*n* = 43)	No burosumab treatment (*n* = 26)	*P*-value	Total (*N* = 77)	Burosumab-treated (*n* = 29)	No burosumab treatment (*n* = 48)	*P*-value
Sex, female	48 (69.6)	31 (72.1)	17 (65.4)	0.557	51 (66.2)	17 (58.6)	34 (70.8)	0.272
Age, years	10.50 (6.00, 14.50)	9.50 (6.75, 14.00)	12.25 (5.25, 14.88)	0.771	37.00 (24.00, 46.50)	38.50 (24.00, 50.50)	35.00 (24.25, 45.13)	0.535
Height, *Z*-score^a^	−1.94 (−2.57, −1.23)	−2.07 (−2.69, −1.60)	−1.51 (−2.32, −0.91)	0.044	−2.01 (−2.60, −1.14)	−1.96 (−2.77, −1.45)	−2.03 (−2.55, −1.09)	0.574
Weight, Z-score^a^	−0.49 (−1.17, 0.37)	−0.49 (−1.36, 0.20)	−0.31 (−0.87, 0.45)	0.220	−0.66 (−1.25, 0.17)	0.11 (−1.00, 0.56)	−0.86 (−1.36, −0.08)	0.017
*PHEX* mutation	36 (52.2)	21 (48.8)	15 (57.7)	0.475	33 (42.9)	11 (37.9)	22 (45.8)	0.497
Intact FGF23, pg/mL	162.50 (102.95, 248.00)	132.50 (95.83, 202.50)	202.00 (140.75, 297.00)	0.032	140.00 (89.80, 437.00)	155.00 (116.00, 412.00)	129.00 (87.55, 454.00)	0.535
Phosphate, mg/dL	2.60 (2.40, 3.03)	2.60 (2.23, 2.90)	2.90 (2.60, 3.10)	0.076	2.30 (2.00, 2.60)	2.10 (1.90, 2.30)	2.40 (2.10, 2.70)	0.043
1,25(OH)_2_D, pg/mL	48.85 (37.65, 67.50)	46.60 (36.45, 58.88)	55.90 (39.33, 71.68)	0.050	44.10 (35.70, 54.60)	44.10 (33.20, 52.50)	44.00 (36.30, 59.93)	0.504
25(OH)D, ng/mL	17.00 (14.00, 22.25)	15.00 (13.25, 17.75)	22.50 (17.25, 24.75)	<0.001	15.00 (13.00, 21.00)	14.00 (12.00, 20.00)	16.00 (13.75, 21.13)	0.097
Calcium, mg/dL	9.50 (9.30, 9.70)	9.45 (9.30, 9.70)	9.55 (9.20, 9.88)	0.829	9.30 (9.10, 9.60)	9.30 (9.10, 9.70)	9.30 (9.08, 9.53)	0.720
iPTH, pg/mL	46.00 (33.00, 63.25)	45.50 (33.00, 62.75)	47.00 (37.25, 63.75)	0.545	61.00 (39.00, 93.00)	76.00 (39.00, 109.00)	54.50 (39.50, 85.50)	0.265
ALP, U/L	450.63 (313.51, 588.88)	459.90 (344.66, 591.76)	446.08 (262.33, 551.51)	0.264	–	–	–	–
BALP, μg/L	–	–	–	–	22.90 (15.80, 37.50)	29.40 (17.90, 48.40)	22.00 (14.80, 34.28)	0.100
Prescription history	–	–	–	>0.999	–	–	–	0.030
Both^b^	67 (97.1)	42 (97.7)	25 (96.2)	–	56 (72.7)	25 (86.2)	31 (64.6)	
Oral phosphate only	0 (0.0)	0 (0.0)	0 (0.0)	–	1 (1.3)	1 (3.4)	0 (0.0)	
Active vitamin D only	0 (0.0)	0 (0.0)	0 (0.0)	–	15 (19.5)	2 (6.9)	13 (27.1)	
Neither	2 (2.9)	1 (2.3)	1 (3.8)	–	5 (6.5)	1 (3.4)	4 (8.3)	

Data are n (%) or median (interquartile range), and include measurements taken >90 days prior to the date of burosumab treatment for the burosumab-treated group and measurements taken >90 days prior to the date of obtaining informed consent for the no burosumab treatment group. Each patient was counted once. Patients who initiated burosumab during the follow-up period were classified as burosumab-treated, and those who did not were classified as non-burosumab treated.

1,25(OH)_2_D, 1,25-dihydroxyvitamin D; 25(OH)D, 25-hydroxyvitamin D; ALP, alkaline phosphatase; BALP, bone-specific alkaline phosphatase; FGF23, fibroblast growth factor 23; iPTH, intact parathyroid hormone. *P*-values indicate results of comparisons between patients with or without burosumab treatment by Pearson's χ^2^ test, Fisher's exact test, or a Mann–Whitney *U* test.

a Z-score was calculated using the following site: http://jspe.umin.jp/medical/chart_dl.html (taikakushisu_v3.3.)

b Both, oral phosphate and active vitamin D.

In children, median (IQR) serum 1,25(OH)_2_D and 25(OH)D were lower at baseline in those who were treated with burosumab than those who were not (46.60 [36.45, 58.88] pg/mL vs 55.90 [39.33, 71.68] pg/mL, *p* = 0.050, and 15.00 [13.25, 17.75] ng/mL vs 22.50 [17.25, 24.75] ng/mL, *p* < 0.001, respectively). No such difference was observed in adults (Table 1).

Median (IQR) baseline serum ALP levels were similar in children who received burosumab (459.90 [344.66, 591.76] U/L) and those who did not (446.08 [262.33, 551.51] U/L, *p* = 0.264). Median (IQR) baseline serum BALP levels tended to be higher in adults treated with burosumab (29.40 [17.90, 48.40] μg/L) than in adults who were not treated with burosumab (22.00 [14.80, 34.28] μg/L, *p* = 0.100).

### 3.2 Changes in serum assessments over time

**Fig. 1:**
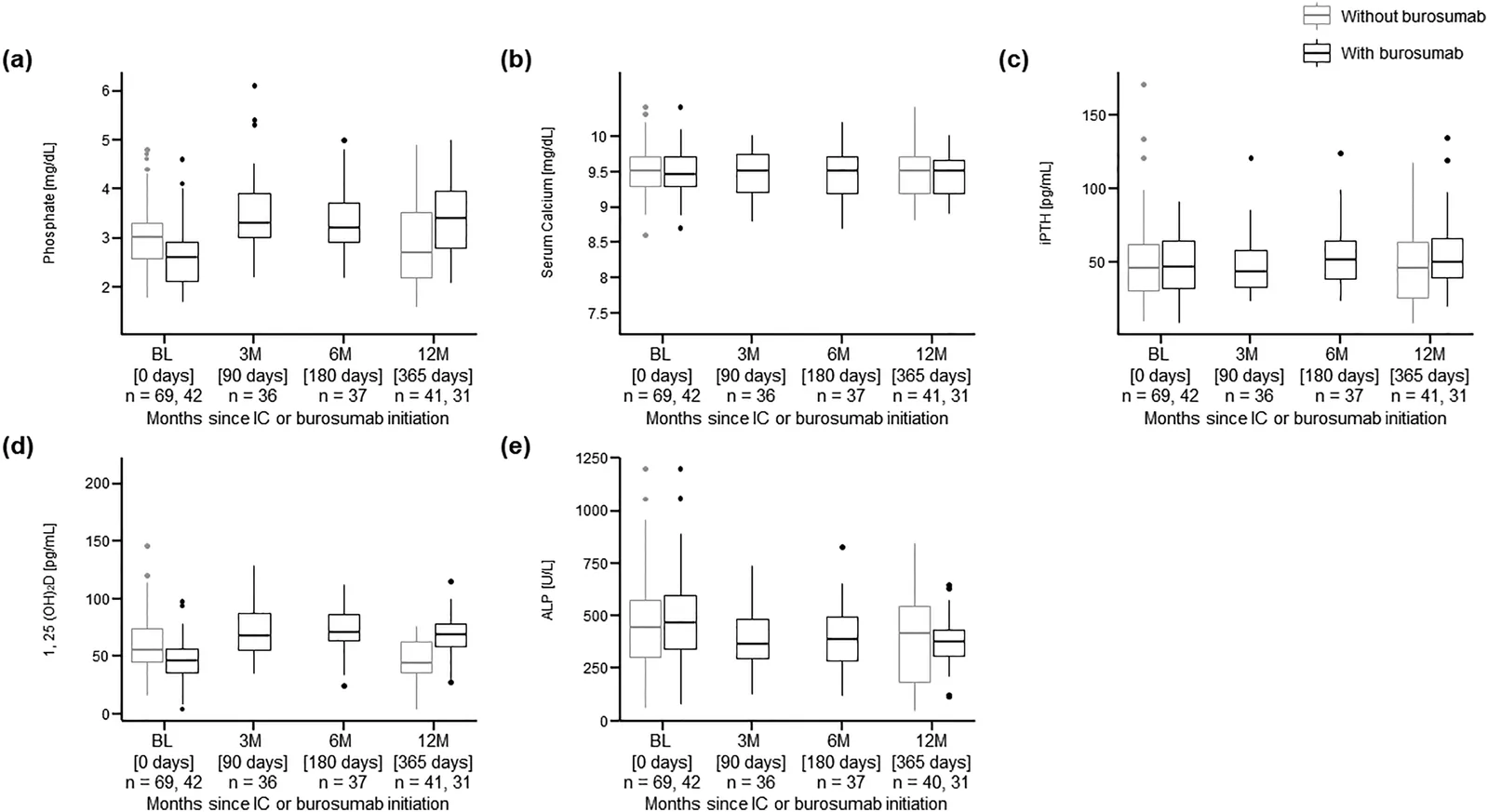
Serum markers at baseline and up to 12 months in children (<18 years). (a) phosphate, (b) serum calcium, (c) parathyroid hormone, (d) 1,25(OH)_2_D, and (e) ALP. Horizontal lines indicate medians, box edges represent interquartile ranges, whiskers show the range of the remaining data within 1.5 times the IQR from the box edges, and data points outside the whisker range are displayed as dots. 1,25(OH)_2_D, 1,25-dihydroxyvitamin D; ALP, alkaline phosphatase; BL, baseline; IC, informed consent; iPTH, intact parathyroid hormone; M, months.

**Fig. 2:**
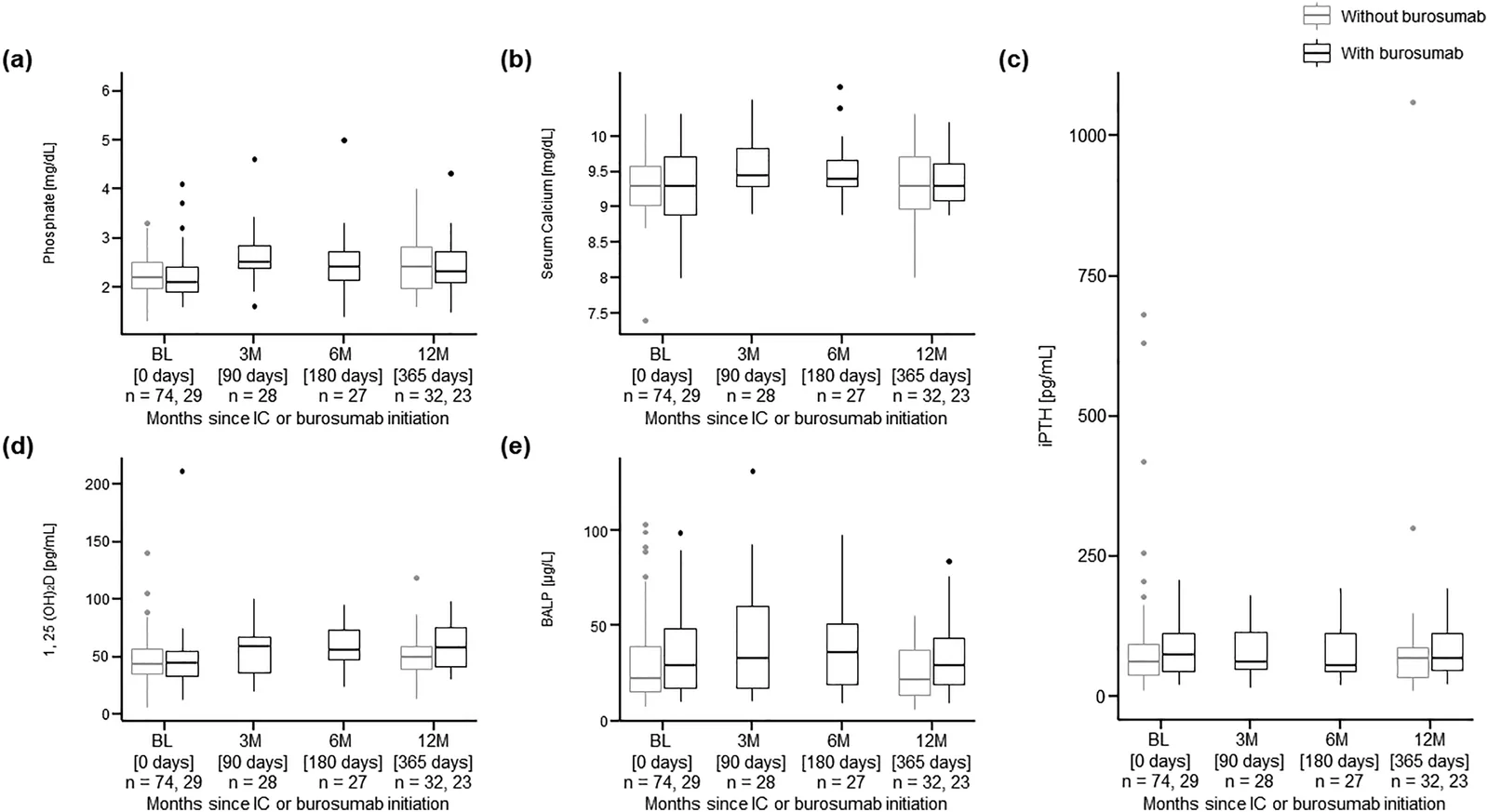
Serum markers at baseline and up to 12 months in adults (≥18 years). (a) phosphate, (b) serum calcium, (c) parathyroid hormone, (d) 1,25(OH)_2_D, and € BALP. Horizontal lines indicate medians, box edges represent interquartile ranges, whiskers show the range of the remaining data within 1.5 times the IQR from the box edges, and data points outside the whisker range are displayed as dots. 1,25(OH)_2_D, 1,25-dihydroxyvitamin D, BALP, bone-specific alkaline phosphatase; BL, baseline; IC, informed consent; iPTH, intact parathyroid hormone; M, months.

Serum evaluations for children and adults who received or did not receive burosumab are shown in Fig. 1 and Fig. 2, respectively. Among children who received burosumab vs those who did not, respective median (IQR) phosphate levels were 2.60 (2.13, 2.90) mg/dL vs 3.00 (2.60, 3.30) mg/dL at baseline and 3.40 (2.80, 3.95) mg/dL vs 2.70 (2.20, 3.50) mg/dL at 1 year. For burosumab-treated children vs non-burosumab-treated children, median iPTH levels were 47.00 (33.00, 63.75) pg/mL vs 46.00 (31.00, 62.00) pg/mL at baseline and 50.00 (40.00, 65.50) pg/mL vs 46.00 (26.00, 64.00) pg/mL at 1 year. Moreover, median 1,25(OH)_2_D levels were 46.20 (36.45, 55.68) pg/mL vs 55.30 (46.00, 73.20) pg/mL at baseline and 68.60 (58.85, 78.30) pg/mL vs 44.30 (36.20, 62.50) pg/mL at 1 year. Median ALP levels were 468.65 (344.66, 591.76) U/L vs 445.20 (302.75, 568.75) U/L at baseline and 379.75 (313.60, 430.33) U/L vs 420.35 (190.49, 542.06) U/L at 1 year. The changes in these parameters for the population treated with burosumab for 1 year and those not treated for 1 year are shown in **Supplementary Fig. S1**.

Among adults who received burosumab vs those who did not, respectively, median (IQR) phosphate levels were 2.10 (1.90. 2.40) mg/dL vs 2.20 (2.00, 2.50) mg/dL at baseline and 2.30 (2.10, 2.70) vs 2.40 (1.98, 2.80) mg/dL at 1 year. For burosumab-treated adults vs non-burosumab-treated adults, median iPTH levels were 76.00 (47.00, 113,00) pg/mL vs 61.50 (40.50, 91.00) pg/mL at baseline and 66.00 (48.50, 110.00) pg/mL vs 69.00 (35.00, 87.00) pg/mL at 1 year. Moreover, median 1,25(OH)_2_D levels were 44.90 (33.70, 53.20) pg/mL vs 42.90 (35.90, 56.50) pg/mL at baseline and 57.90 (40.75, 74.50) pg/mL vs 49.90 (39.48, 58.15) pg/mL at 1 year. Median BALP was 29.40 (17.30, 48.40) μg/L vs 22.15 (15.65, 38.85) μg/L at baseline and 28.90 (19.45, 43.15) μg/L vs 21.30 (13.60, 37.13) μg/L at 1 year. The changes in these parameters for the population treated with burosumab for 1 year and those not treated for 1 year are shown in **Supplementary Fig. S2**.

### 3.3 Proportion of patients in the target range for phosphate, ALP, and BALP

The percentage of children who did not receive burosumab who were in the target range for phosphate was 15.9% (11/69) at baseline and 12.2% (5/41) at 1 year, and the percentage who were in the target range for ALP was 31.9% (22/69) at baseline and 37.5% (15/40) at 1 year (Table 2). In the burosumab-treated group, the percentage of children in the target range at baseline was 4.8% (2/42) for phosphate and 26.2% (11/42) for ALP. After 1 year of burosumab treatment, the percentage of children in the target range for phosphate was 19.4% (6/31), and the percentage in the target range for ALP was 41.9% (13/31).

**Table 2:** Serum phosphate and bone metabolism markers before treatment and 12 months after burosumab treatment or informed consent.

	All patients				Patients treated for full 12 months			
	No burosumab treatment		Burosumab-treated		No burosumab treatment		Burosumab-treated	
	Baseline	12 months	Baseline	12 months	Baseline	12 months	Baseline	12 months
**Children (<18 years)**								
Phosphate (mg/dL), n	69	41	42	31	40	41	31	31
Median (IQR)	3.00 (2.60, 3.30)	2.70 (2.20, 3.50)	2.60 (2.13, 2.90)	3.40 (2.80, 3.95)	3.05 (2.60, 3.50)	2.70 (2.20, 3.50)	2.50 (2.15, 3.00)	3.40 (2.80, 3.95)
Within target range								
Yes (≥LLN)	11 (15.9)	5 (12.2)	2 (4.8)	6 (19.4)	9 (22.5)	5 (12.2)	2 (6.5)	6 (19.4)
No (<LLN mg/L)	58 (84.1)	36 (87.8)	40 (95.2)	25 (80.6)	31 (77.5)	36 (87.8)	29 (93.5)	25 (80.6)
ALP(U/L), n	69	40	42	31	40	40	31	31
Median (IQR)	445.20 (302.75, 568.75)	420.35 (190.49, 542.06)	468.65 (344.66, 591.76)	379.75 (313.60, 430.33)	365.75 (201.69, 525.44)	420.35 (190.49, 542.06)	536.55 (420.35, 639.10)	379.75 (313.60, 430.33)
Within the target range								
Yes (≤ULN)	22 (31.9)	15 (37.5)	11 (26.2)	13 (41.9)	17 (42.5)	15 (37.5)	6 (19.4)	13 (41.9)
No (>ULN)	47 (68.1)	25 (62.5)	31 (73.8)	18 (58.1)	23 (57.5)	25 (62.5)	25 (80.6)	18 (58.1)
**Adults (≥18 years)**								
Phosphate (mg/dL), n	74	32	29	23	32	32	23	23
Median (IQR)	2.20 (2.00, 2.50)	2.40 (1.98, 2.80)	2.10 (1.90, 2.40)	2.30 (2.10, 2.70)	2.35 (2.10, 2.70)	2.40 (1.98, 2.80)	2.10 (1.90, 2.35)	2.30 (2.10, 2.70)
Within target range								
Yes (≥2.5 mg/dL)	22 (29.7)	15 (46.9)	7 (24.1)	9 (39.1)	14 (43.8)	15 (46.9)	5 (21.7)	9 (39.1)
No (<2.5 mg/dL)	52 (70.3)	17 (53.1)	22 (75.9)	14 (60.9)	18 (56.3)	17 (53.1)	18 (78.3)	14 (60.9)
**Female adults (≥18 years)**								
BALP (μg/L), n	48	20	17	13	20	20	13	13
Median (IQR)	18.60 (13.33, 26.78)	19.85 (11.93, 29.98)	22.90 (15.90, 34.30)	20.90 (13.90, 40.80)	19.30 (12.23, 27.80)	19.85 (11.93, 29.98)	22.90 (16.30, 34.30)	20.90 (13.90, 40.80)
Within target range								
Yes (≤14.5 μg/L)	14 (29.2)	8 (40.0)	4 (23.5)	4 (30.8)	8 (40.0)	8 (40.0)	2 (15.4)	4 (30.8)
No (>14.5 μg/L)	34 (70.8)	12 (60.0)	13 (76.5)	9 (69.2)	12 (60.0)	12 (60.0)	11 (84.6)	9 (69.2)
**Male adults (≥18 years)**								
BALP (μg/L), n	26	12	12	10	12	12	10	10
Median (IQR)	39.50 (21.05, 61.00)	36.30 (20.30, 42.53)	39.40 (21.00, 49.53)	31.25 (27.70, 44.23)	41.85 (26.35, 63.28)	36.30 (20.30, 42.53)	42.70 (33.85, 50.98)	31.25 (27.70, 44.23)
Within target range								
Yes (≤20.9 μg/L)	7 (26.9)	3 (25.0)	3 (25.0)	0 (0.0)	3 (25.0)	3 (25.0)	2 (20.0)	0 (0.0)
No (>20.9 μg/L)	19 (73.1)	9 (75.0)	9 (75.0)	10 (100.0)	9 (75.0)	9 (75.0)	8 (80.0)	10 (100.0)

Data are n or n (%), unless otherwise stated. The baseline values include measurements taken up to 90 days prior to the baseline. Baseline values for patients who did not receive burosumab treatment included patients from both the burosumab-treated group (prior to receiving burosumab) and the non-burosumab-treated group. Patients with missing values or values measured outside of the allowed time period were omitted.

ALP, alkaline phosphatase; BALP, bone-specific alkaline phosphatase; IQR, interquartile range.

For adults who did not receive burosumab, 29.7% (22/74) at baseline and 46.9% (15/32) at 1 year were in the target range for phosphate (Table 2). In burosumab-treated adults, the percentage in the target range for phosphate was 24.1% (7/29) before initiating burosumab treatment and 39.1% (9/23) after 1 year of burosumab treatment. For female adults who did not receive burosumab, the percentage in the target range for BALP was 29.2% (14/48) at baseline and 40.0% (8/20) at 1 year. For female adults treated with burosumab, the percentage in the target range for BALP was 23.5% (4/17) before burosumab treatment and 30.8% (4/13) after 1 year of burosumab treatment. No male adults (*n* = 10) were in the target range for BALP after 1 year of burosumab treatment.

When only patients who received treatment for 1 year were analyzed, the percentage of children who did not receive burosumab and who were in the target range for phosphate was 22.5% (9/40) at baseline and 12.2% (5/41) at 1 year, and the percentage who were in the target range for ALP was 42.5% (17/40) at baseline and 37.5% (15/40) at 1 year (Table 2). For burosumab-treated children, the percentage who were in the target range for phosphate was 6.5% (2/31) at baseline and 19.4% (6/31) at 1 year, and the percentage who were in the target range for ALP was 19.4% (6/31) at baseline and 41.9% (13/31) at 1 year. Among adults who did not receive burosumab, the percentage of patients in the target range for phosphate was 43.8% (14/32) at baseline and 46.9% (15/32) at 1 year. For burosumab-treated adults, the percentage in the target range for phosphate was 21.7% (5/23) before initiating burosumab treatment and 39.1% (9/23) after 1 year of burosumab treatment. Among female adults who did not receive burosumab, the percentage in the target range for BALP was 40.0% (8/20) at baseline and 40.0% (8/20) at 1 year. For burosumab-treated female adults, the percentage in the target range for BALP was 15.4% (2/13) before burosumab treatment and 30.8% (4/13) after 1 year of burosumab treatment. No male adults (*n* = 10) were in the target range for BALP after 1 year of burosumab treatment.

### 3.4 Change in height over time

**Fig. 3:**
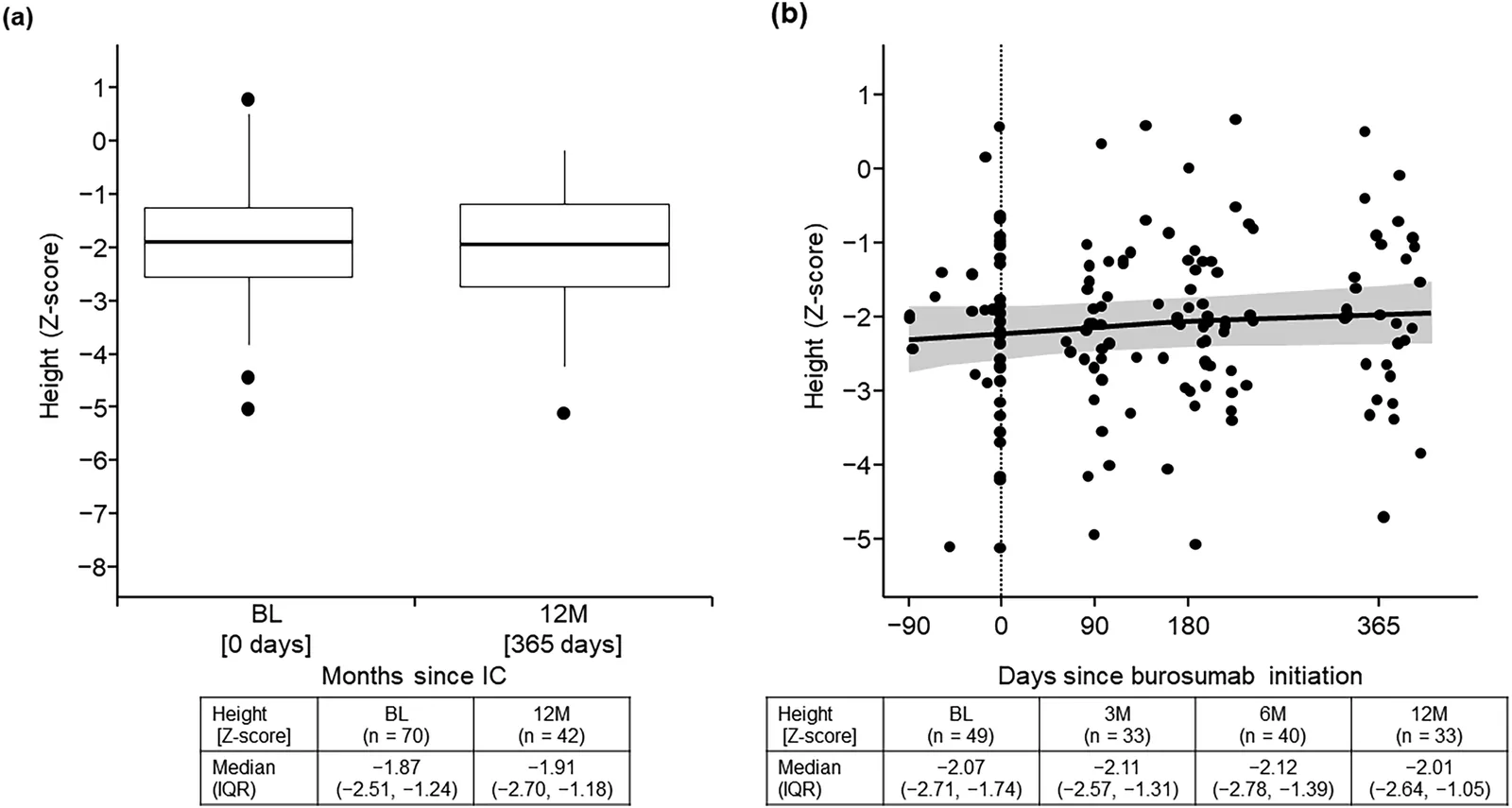
Change in height *Z*-scores over time in children (<18 years). (a) Patients not treated with burosumab. Horizontal lines indicate medians, box edges represent interquartile ranges, whiskers show the range of the remaining data within 1.5 times the IQR from the box edges, and data points outside the whisker range are displayed as dots. (b) Patients treated with burosumab. Data are individual datapoints, with the horizontal line indicating mean values and shaded gray areas representing SDs. BL, baseline; IC, informed consent; IQR, interquartile range; M, months.

Among children who were not treated with burosumab, the median (IQR) height *Z*-score at baseline was −1.87 (−2.51, −1.24) and at 1 year was −1.91 (−2.70, −1.18) (Fig. 3A). In burosumab-treated children, the median height *Z*-score before treatment was −2.07 (−2.71, −1.74). After 3 months, 6 months, and 1 year of burosumab treatment, the median height Z-score was −2.11 (−2.57, −1.31), −2.12 (−2.78, −1.39), and −2.01 (−2.64, −1.05), respectively (Fig. 3B).

### 3.5 Association of rickets severity and height with ALP

**Fig. 4:**
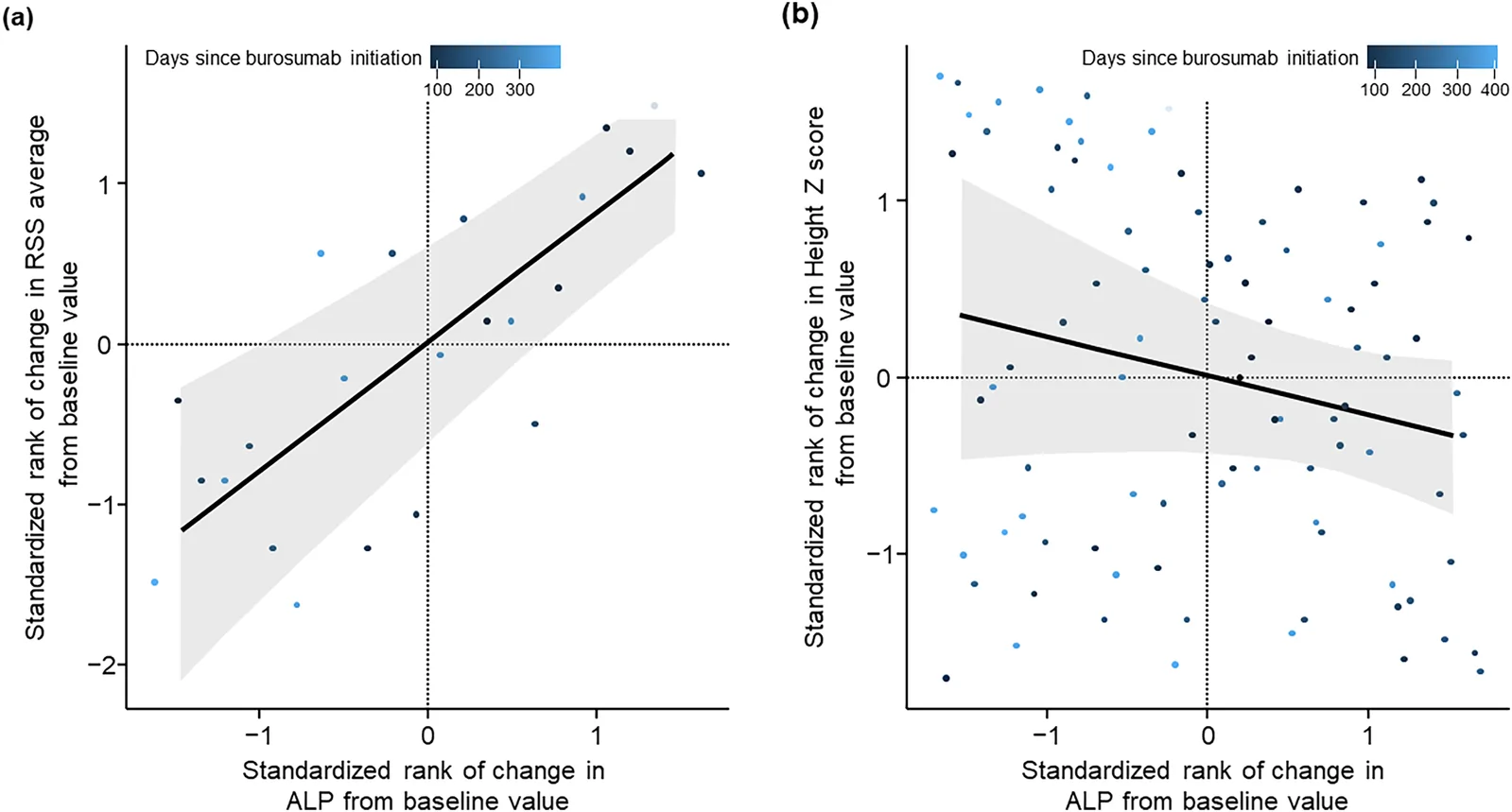
Correlation between height and RSS and bone metabolism markers in children (<18 years) treated with burosumab. (a) Correlation between RSS and ALP in children. Data are individual datapoints, with the horizontal lines indicating mean values and shaded gray areas representing SDs. (b) Correlation between change in height Z-score and ALP in children. Data are individual datapoints, with the horizontal lines indicating mean values and shaded gray areas representing SDs. ALP, alkaline phosphate; RSS, rickets severity score.

Among children, the median (IQR) RSS was 2.88 (1.72, 3.69) before burosumab initiation and decreased to 1.13 (0.72, 1.19) after 1 year of burosumab treatment (Table 3). The correlation between the standardized change from baseline in RSS or height and ALP is shown in Fig. 4. RSS changes significantly correlated with ALP changes (correlation coefficient: 0.806 [95% CI: 0.540, 1.073], *p* < 0.001), and the average height Z-score changes tended to correlate with ALP changes (correlation coefficient: −0.219 [−0.531, 0.092], *p* = 0.165) (Table 4).

**Table 3:** Outcome changes before burosumab treatment and 6 months and 1 year after burosumab initiation.

	Before burosumab initiation	6 months	1 year
**Children (<18 years)**			
6MWT, m	37	29	24
	447.00 (374.00, 495.00)	440.00 (385.00, 484.00)	460.00 (402.50, 496.50)
SF10 PSS score	38	30	27
	51.59 (48.91, 56.93)	51.59 (48.91, 56.93)	51.59 (47.58, 54.26)
SF10 PHS score	38	30	27
	48.02 (42.96, 53.81)	48.36 (44.95, 53.81)	50.40 (46.77, 53.81)
EQ-5D-Y	19	15	14
	0.98 (0.98, 0.98)	0.98 (0.98, 0.98)	0.98 (0.98, 0.98)
EQ-VAS-Y	19	15	14
	90.00 (70.00, 100.00)	100.00 (82.50, 100.00)	90.00 (81.25, 98.75)
FPS-R	38	30	27
	1.00 (1.00, 2.00)	1.00 (1.00, 1.00)	1.00 (1.00, 1.50)
Days unable to attend school because of XLH symptoms/complications	33	–	21
	0.00 (0.00, 0.00)	–	0.00 (0.00, 0.00)
Days unable to attend school because of managing XLH	33	–	21
	1.00 (0.00, 5.00)	–	1.00 (0.00, 6.00)
RSS	16	7	8
	2.88 (1.72, 3.69)	1.13 (0.44, 1.38)	1.13 (0.72, 1.19)
RGI-C	0	5	7
	NA	1.00 (0.33, 1.67)	1.00 (1.00, 1.50)
PROMIS proxy (age: 5–7 years) T-score - Mobility	9	7	6
	45.50 (43.20, 57.20)	57.20 (53.40, 57.20)	57.20 (48.65, 57.20)
PROMIS proxy (age: 5–7 years) T-score - Fatigue	9	7	6
	41.00 (38.90, 45.60)	38.90 (35.20, 43.90)	37.05 (35.20, 41.68)
PROMIS proxy (age: 5–7 years) T-score - Pain interference	9	7	6
	39.90 (39.90, 50.30)	39.90 (39.90, 45.95)	39.90 (39.90, 39.90)
PROMIS (age: 8–18 years) T-score - Mobility	29	23	21
	59.10 (53.70, 59.10)	59.10 (59.10, 59.10)	59.10 (50.70, 59.10)
PROMIS (age: 8–18 years) T-score - Fatigue	29	23	21
	36.80 (32.40, 40.70)	36.50 (32.40, 39.60)	32.40 (32.40, 39.00)
PROMIS (age: 8–18 years) T-score - Pain interference	29	23	21
	36.80 (36.80, 41.40)	36.80 (36.80, 42.25)	36.80 (36.80, 36.80)
**Adults (≥18 years)**			
TUG, s	29	27	23
	10.07 (8.86, 13.10)	9.88 (8.63, 11.19)	9.56 (8.97, 12.90)
Average Grip Strength, kg	33	27	23
	26.00 (23.00, 30.00)	27.00 (22.50, 30.00)	27.00 (21.50, 31.00)
EQ-5D-5L	30	27	23
	0.66 (0.48, 0.94)	0.94 (0.57, 0.94)	0.66 (0.48, 0.94)
EQ-VAS	30	27	23
	70.00 (60.00, 80.00)	80.00 (62.50, 89.00)	80.00 (65.00, 90.00)
BPI worst pain	30	27	23
	3.50 (1.25, 6.00)	3.00 (0.50, 6.00)	3.00 (1.00, 6.00)
BPI least pain	30	27	23
	0.00 (0.00, 1.00)	0.00 (0.00, 1.00)	0.00 (0.00, 2.00)
BPI average pain	30	27	23
	2.00 (1.00, 4.00)	2.00 (0.00, 3.50)	2.00 (0.50, 4.00)
BPI now pain	30	27	23
	1.00 (0.00, 3.00)	1.00 (0.00, 3.00)	1.00 (0.00–3.00)
BPI pain severity	30	27	23
	2.00 (0.81, 3.19)	1.50 (0.13, 3.25)	2.25 (0.50, 3.63)
BPI pain interference	29	27	23
	2.00 (0.00, 4.00)	0.86 (0.00, 3.57)	2.57 (0.43, 4.86)
WOMAC subscale score, maximum pain	29	27	23
	25.00 (10.00, 35.00)	15.00 (2.50, 30.00)	25.00 (12.50, 37.50)
WOMAC subscale score, maximum stiffness	29	27	23
	25.00 (0.00, 50.00)	12.50 (0.00, 37.50)	25.00 (12.50, 50.00)
WOMAC subscale score, maximum physical function	29	27	23
	17.65 (1.47, 32.35)	11.76 (3.68, 29.41)	11.76 (6.62, 33.82)
Days unable to attend work because of XLH symptoms/complications	16	–	14
	0.00 (0.00, 1.25)	–	0.00 (0.00, 0.00)
Days unable to attend work because of managing XLH	16	–	14
	3.50 (0.00, 10.50)	–	3.00 (0.00, 11.50)
PROMIS (age: 18 years) T-score - Physical function	17	25	23
	30.10 (23.10, 36.20)	29.30 (15.50, 33.50)	28.60 (23.75, 33.50)

Data are n or median (interquartile range).

6MWT, 6-min walk test; BPI, Brief Pain Inventory; EQ-5D-5L, EQ-5D five-level version; EQ-5D-Y, EQ-5D child version; EQ-VAS, EQ visual analogue scale; EQ-VAS-Y, EQ visual analogue scale child version; FPS-R, Faces Pain Scale revised; IQR, interquartile range; NA, not applicable; PHS, Physical Summary Scale; PPS, Palliative Performance Scale; PROMIS, Patient-Reported Outcomes Measurement Information System; RGI-C, Radiographical Global Impression of Change; RSS, Rickets Severity Score; SF10, 10-item Short Form health survey; TUG, Timed Up & Go test; XLH, X-linked hypophosphatemia.

**Table 4:** Correlation between outcome changes and bone metabolism markers.

Children (<18 years)	Correlation coefficient with ALP (95% CI)	*P*-value
Height Z-score	−0.219 (−0.531, 0.092)	0.165
6MWT	0.268 (−0.119, 0.655)	0.170
SF10 PSS score	0.138 (−0.244, 0.520)	0.471
SF10 PHS score	−0.051 (−0.429, 0.328)	0.790
EQ-5D-Y	−0.013 (−0.683, 0.657)	0.969
EQ-VAS-Y	−0.115 (−0.693, 0.463)	0.684
FPS-R	0.180 (−0.142, 0.501)	0.267
Days unable to attend school because of XLH symptoms/complications	−0.113 (−0.728, 0.501)	0.703
Days unable to attend school because of managing XLH	−0.238 (−0.788, 0.312)	0.375
RSS	0.806 (0.540, 1.073)	<0.001
RGI-C	−0.168 (−0.881, 0.544)	0.622
PROMIS proxy (age: 5–7 years) T-score		
Mobility	−0.115 (−0.820, 0.591)	0.718
Fatigue	0.274 (−0.290, 0.838)	0.295
Pain interference	0.075 (−0.571, 0.722)	0.795
PROMIS (age: 8–18 years) T-score		
Mobility	0.169 (−0.351, 0.688)	0.516
Fatigue	0.052 (−0.438, 0.541)	0.832
Pain interference	0.057 (−0.400, 0.515)	0.801


Adults (≥18 years)	Correlation coefficient with BALP (95% CI)	*P*-value
TUG	−0.090 (−0.476, 0.296)	0.643
Average Grip Strength	−0.313 (−0.625, 0.000)	0.050
EQ-5D-5L	−0.182 (−0.517, 0.153)	0.279
EQ-VAS	−0.019 (−0.360, 0.323)	0.913
BPI		
Worst pain	0.146 (−0.208, 0.500)	0.410
Least pain	−0.161 (−0.536, 0.214)	0.392
Average pain	−0.026 (−0.417, 0.366)	0.896
Now pain	0.000 (−0.380, 0.380)	1.000
Pain severity	0.038 (−0.349, 0.425)	0.845
Pain interference	0.157 (−0.177, 0.491)	0.349
WOMAC subscale score		
Maximum pain	0.113 (−0.218, 0.443)	0.497
Maximum stiffness	0.054 (−0.196, 0.305)	0.664
Maximum physical function	0.245 (−0.095, 0.586)	0.154
Days unable to attend work because of XLH symptoms/complications	−0.186 (−0.650, 0.278)	0.411
Days unable to attend work because of managing XLH	−0.212 (−0.658, 0.234)	0.333
PROMIS (age: 18 years) T-score - Physical function	−0.208 (−0.555, 0.139)	0.234

6MWT, 6-min walk test; ALP, alkaline phosphatase; BALP, bone-specific alkaline phosphatase; BPI, Brief Pain Inventory; CI, confidence interval; EQ-5D-5L, EQ-5D five-level version; EQ-5D-Y, EQ-5D child version; EQ-VAS, EQ visual analogue scale; EQ-VAS-Y, EQ visual analogue scale child version; FPS-R, Faces Pain Scale revised; PHS, Physical Summary Scale; PPS, Palliative Performance Scale; PROMIS, Patient-Reported Outcomes Measurement Information System; RGI-C, Radiographical Global Impression of Change; RSS, Rickets Severity Score; SF10, 10-item Short Form health survey; TUG, Timed Up & Go test; XLH, X-linked hypophosphatemia.

### 3.6 Association of motor function and QoL/PROs with ALP/BALP

In children, the median (IQR) 6-min walk test result was 447.00 (374.00, 495.00) m prior to burosumab treatment and 460.00 (402.50, 496.50) m after 1 year of treatment. In adults, the median (IQR) Timed Up & Go test time was 10.07 (8.86, 13.10) seconds before burosumab treatment and 9.56 (8.97, 12.90) seconds after 1 year of burosumab treatment (Table 3). Other PRO/QoL changes are listed in Table 3. There were no significant correlations between any QoL/PRO assessments and ALP/BALP (Table 4).

### 3.7 Burosumab dosage

**Fig. 5:**
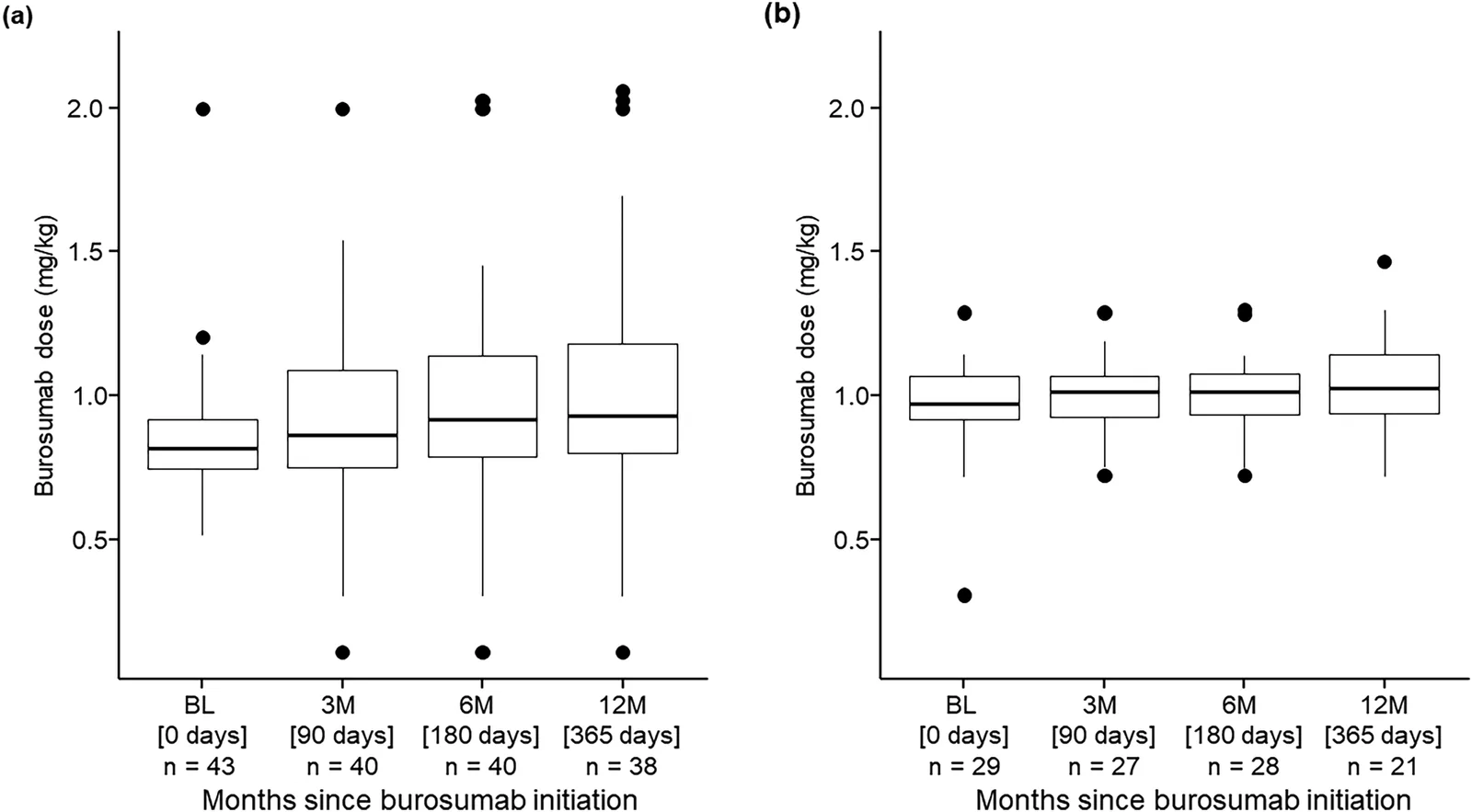
Burosumab dose over time in (a) children (<18 years) and (b) adults (≥18 years). Horizontal lines indicate medians, box edges represent interquartile ranges, whiskers show the range of the remaining data within 1.5 times the IQR from the box edges, and data points outside the whisker range are displayed as dots. BL, baseline; M, months.

The dose over time in patients treated with burosumab is shown in Fig. 5. In children, the burosumab dose tended to increase over time (Fig. 5A), and 42.1% (16/38) of these patients received doses ≥1.0 mg/kg after 1 year of burosumab treatment.

### 3.8 Renal calcification grade

**Fig. 6:**
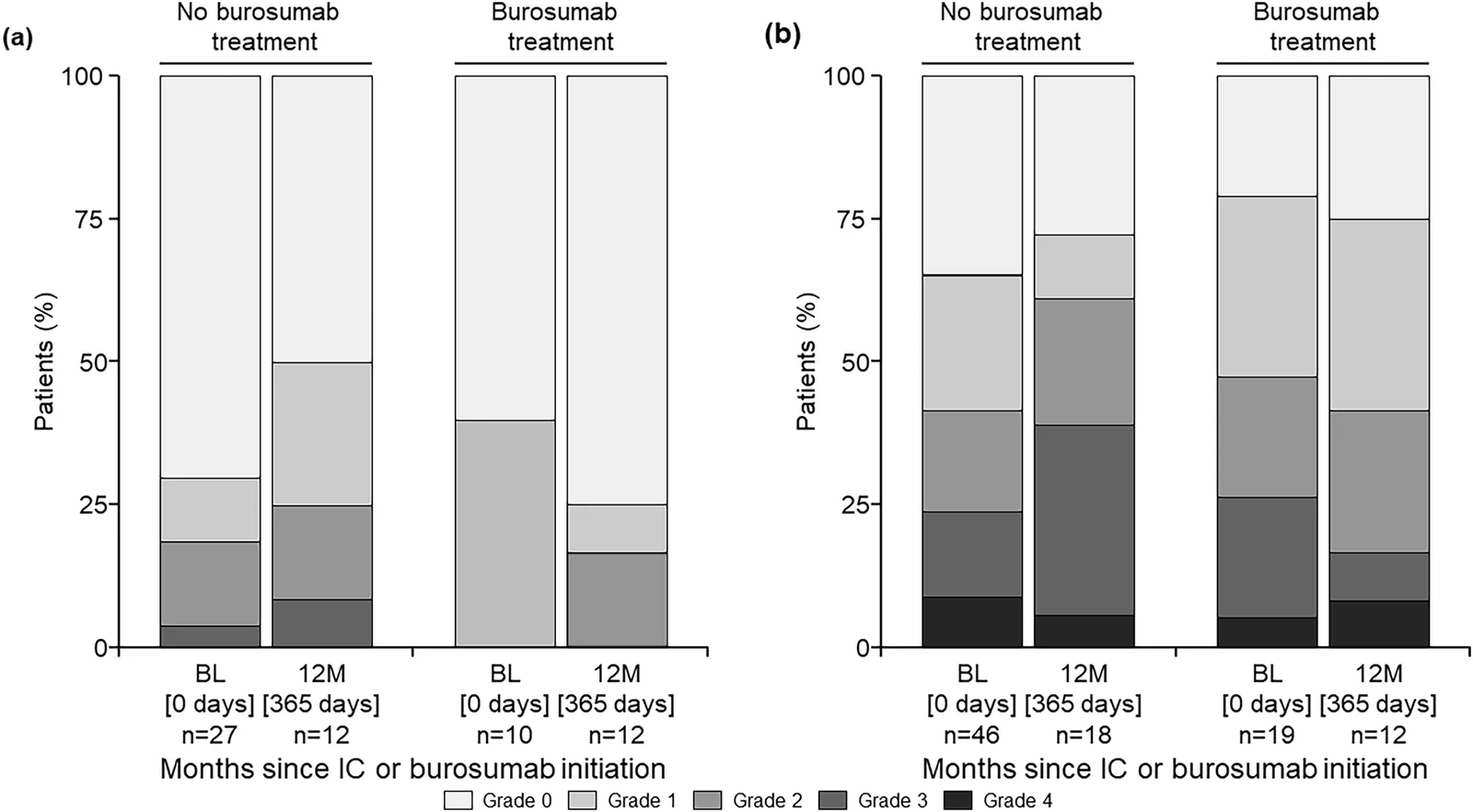
Renal calcification grade changes in (a) children (<18 years) and (b) adults (≥18 years). BL, baseline; IC, informed consent, M, months.

Among children, 6/10 (60.0%) had grade 0 and 4/10 (40.0%) had grade 1 renal calcification before burosumab treatment (Fig. 6A). After 1 year of burosumab treatment, 9/12 (75.0%) had grade 0, 1/12 (8.3%) had grade 1, and 2/12 (16.7%) had grade 2 renal calcification. In adults, 4/19 (21.1%) had grade 0, 6/19 (31.6%) had grade 1, 4/19 (21.1%) had grade 2, 4/19 (21.1%) had grade 3, and 1/19 (5.3%) had grade 4 renal calcification before burosumab treatment (Fig. 6B). After 1 year of burosumab treatment, 3/12 (25.0%), 4/12 (33.3%), 3/12 (25.0%), 1/12 (8.3%), and 1/12 (8.3%) had grade 0, 1, 2, 3, and 4 renal calcification, respectively.

Among children who did not receive burosumab, 19/27 (70.4%) had grade 0, 3/27 (11.1%) had grade 1, 4/27 (14.9%) had grade 2, and 1/27 (3.7%) had grade 3 renal calcification at baseline (Fig. 6A). After 1 year, 6/12 (50.0%) had grade 0, 3/12 (25.0%) had grade 1, 2/12 (16.7%) had grade 2, and 1/12 (8.3%) had grade 3 renal calcification. In adults, 16/46 (34.8%) had grade 0, 11/46 (23.9%) had grade 1, 8/46 (17.4%) had grade 2, 7/46 (15.2%) had grade 3, and 4/46 (8.7%) had grade 4 renal calcification at baseline (Fig. 6B). After 1 year, 5/18 (27.7%) had grade 0, 2/18 (11.1%) had grade 1, 4/18 (22.2%) had grade 2, 6/18 (33.3%) had grade 3, and 1/18 (5.6%) had grade 4 renal calcification.

## 4 Discussion

**Fig. 7:**
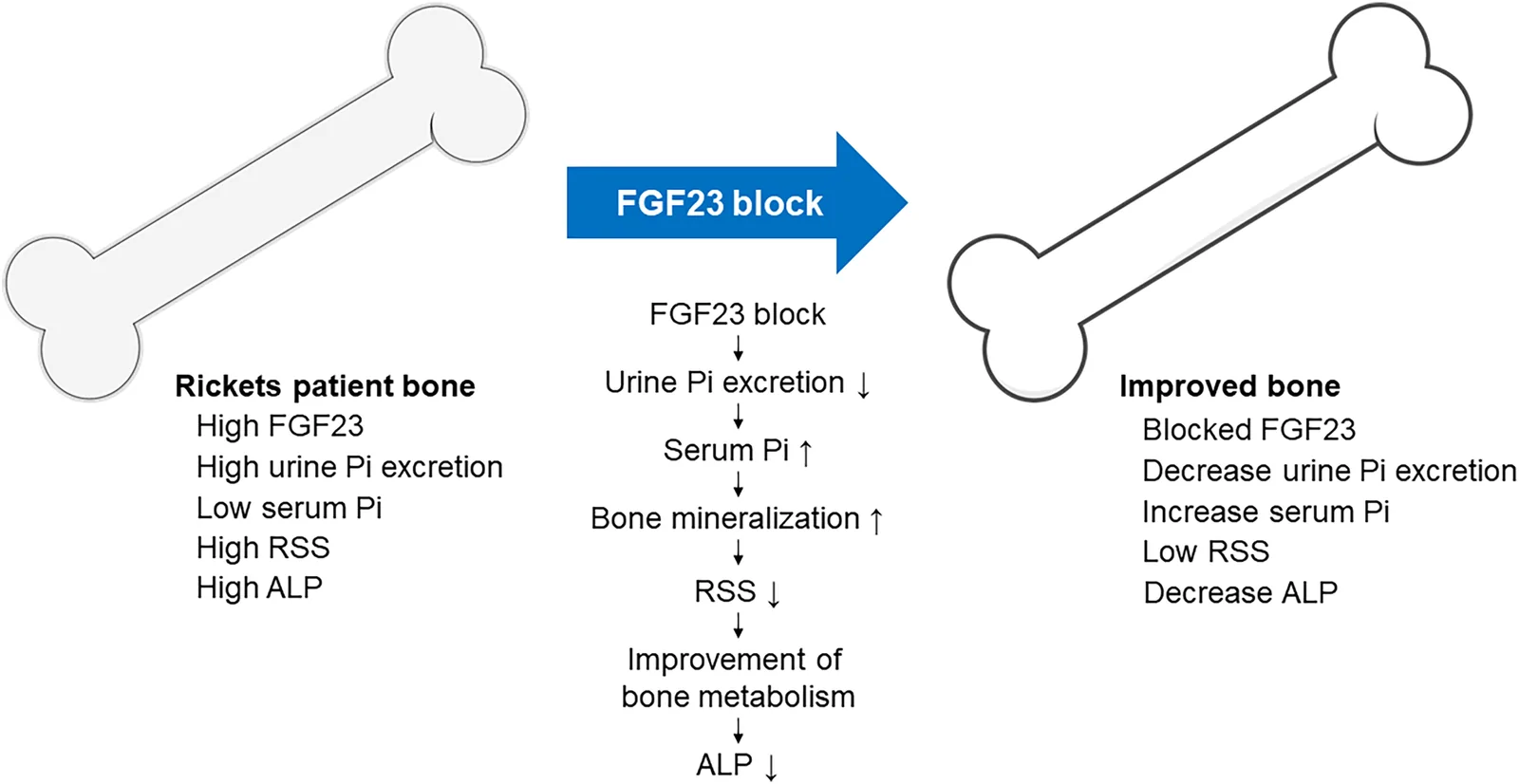
Summary of the effects of burosumab ALP, alkaline phosphate; FGF23, fibroblast growth factor 23; RSS, rickets severity score.

This report describes the 3-year cut-off data of the SUNFLOWER study and includes data of the first year of burosumab administration. The relationships between bone metabolism markers and rickets severity, height, QoL/PROs, and motor function in burosumab-treated patients with XLH were also assessed. The observed effects of burosumab are summarized in Fig. 7. Overall, this analysis showed that treatment with burosumab appeared to improve serum phosphate and bone metabolism markers over 1 year.

In this analysis, burosumab-treated patients had improved serum phosphate levels, 1,25(OH)_2_D levels, and bone metabolism markers (ALP and BALP) at 1 year compared with pre-treatment levels. However, serum phosphate levels in burosumab-treated adults did not increase as much as in previous studies (Ward et al., 2024; Weber et al., 2022; Dodamani et al., 2024). This may be because serum phosphate is often measured immediately before the next dose of burosumab, which often coincides with trough levels (Imel, 2021; Weber et al., 2022; Lee et al., 2022). Additionally, we did not observe any notable difference in the increase in phosphate levels between adults in the burosumab group and those in the non-burosumab group in the all-patients analysis. This may be because data from patients before they initiated burosumab treatment were included in the non-burosumab group, while data from patients who started receiving burosumab within 1 year after baseline were not included in the non-burosumab group at 1 year. Thus, the baseline non-burosumab group included patients who subsequently received burosumab. At baseline, phosphate levels tended to be lower in patients who received burosumab than those who did not receive burosumab, potentially suggesting that burosumab was administered to patients with lower phosphate levels. It is possible that the non-burosumab group may have had lower phosphate levels at baseline because of the inclusion of patients who subsequently received burosumab. Additionally, serum phosphate levels did not increase in adults treated for 1 year in the non-burosumab group, whereas they did increase in the burosumab group. Overall, these findings confirm that an increase in serum phosphate levels occurs following burosumab treatment.

The proportion of patients in the target range for phosphate and ALP increased over the study period in burosumab-treated children. In non-burosumab-treated children, the proportion in the target range for phosphate and ALP did not change or even increased in the all-patients analysis; however, it decreased in patients treated for 1 year. This difference suggests that patients who switched to burosumab during the study had worse baseline biochemical parameters and highlights the difficulty of maintaining biochemical parameters within the normal range with oral phosphate/active vitamin D treatments. Nevertheless, some patients remained outside the target range after 1 year of treatment.

Our results are consistent with previous studies that showed while burosumab improves phosphate levels and ALP levels, some will not return to normal levels despite treatment (Walker et al., 2023; Ewert et al., 2023), suggesting that some patients may require increased doses of burosumab. Furthermore, some patients with XLH experience symptom improvement despite not reaching the target phosphate ranges (Walker et al., 2023), suggesting that treatment efficacy should be evaluated comprehensively and not based solely on phosphate levels. Additionally, the proportion of children achieving the target range was lower in this study than in previous reports (Ward et al., 2024; Imel et al., 2019), which may be because of differences in how target ranges for phosphate and ALP were determined.

In this study, target ranges were age-specific (Colantonio et al., 2012), whereas some earlier studies used unified values across ages (Linglart et al., 2022). Among adults, the proportion of patients in the target range for phosphate also increased in the burosumab group. For patients who did not receive burosumab, the proportion who achieved the target phosphate range also increased in the all-patients analysis but not among those treated for 1 year, similar to the results observed in children. Additionally, the proportion of patients in the target BALP range increased over time in female adults but decreased in male adults. Thus, it may take longer than 1 year for some patients to achieve normal BALP levels, particularly as BALP increases transiently after the administration of burosumab (Insogna et al., 2019; Weber et al., 2022).

Among children who received burosumab treatment for 1 year, we observed improvements in RSS but no notable improvement in height. Improvements in RSS and ALP were significantly correlated, indicating that decreasing ALP is associated with an improvement in rickets. As ALP is a marker for bone turnover (Linglart et al., 2014; Ewert et al., 2023), decreases in ALP would be expected to correlate with RSS improvements. Given that few studies have reported correlations in changes of clinical endpoints with ALP/BALP (Thacher et al., 2019), this negative correlation between ALP and improvements in RSS is notable. However, as no notable improvements in height were observed, our findings contrast with previous studies that reported that height is correlated with rickets severity and ALP in children (Mäkitie et al., 2003; Rajah et al., 2008). Although our results suggested a trend towards improvement in height in patients with decreased ALP, no clear correlation was observed, which may be because of the small change in height *Z*-score. While one previous study reported an improvement in height Z-scores with burosumab treatment (Imel et al., 2019), another did not find similar improvements (Namba et al., 2022). It is possible that the absence of a significant improvement may reflect the inclusion of some patients with mild symptoms. Further analysis beyond 1 year may be necessary to identify improvements in height and any potential association with other outcomes.

In this study, patients who received burosumab tended to have characteristics indicating more severe disease than patients who were not treated with burosumab, as demonstrated by significantly lower baseline serum phosphate levels in adults and significantly lower baseline serum 1,25(OH)_2_D levels in children who received burosumab. However, after 1 year of burosumab treatment, there were no clear improvements in QoL/PROs in children. This may be because patients do not recognize their QoL as being severely impaired at the time of initiating treatment, making it difficult to detect improvements in this population (Ferizović et al., 2020). In adults, some QoL/PROs such as BPI worst pain and WOMAC physical functions appeared to improve over time. BALP also decreased, particularly among female adults. However, no significant correlation between QoL/PROs and BALP improvements was identified, so further follow-up data are needed to clarify this. Moreover, motor function assessments, including the 6-min walk test and the Timed Up & Go test, showed a trend towards improvement over time, but were not correlated with ALP/BALP. Thus, despite burosumab-treated patients in this study appearing to have markers of more severe disease, the observation period for this analysis might not have been sufficient to reveal improvements in QoL/PROs in some patients.

Additionally, renal calcification grades worsened for some patients who did not receive burosumab; however, consistent changes were not observed in the renal calcification grades over the year of burosumab treatment. Pi/D treatment for XLH can lead to worsened renal calcification in patients with XLH (Harada et al., 2021). A recent study examined the development of nephrocalcinosis in children with XLH (Paloian et al., 2024); the effect of burosumab treatment on long-term renal calcification grades remains unclear. However, that study noted that nephrocalcinosis can occur several years after stopping phosphate supplementation. A recent interim analysis of the post-authorization safety study for the International XLH Registry reported that there were no cases of nephrocalcinosis in patients aged <18 years following 2 years of burosumab treatment (Boot et al., 2024). In the present study, some children who were categorized as having grade 0 or 1 renal calcification at baseline were recategorized as grade 2 after 1 year of burosumab treatment. However, the role of burosumab in the development of renal calcification in these patients remains unclear.

Although the dose of burosumab tended to increase over time in children, most were treated with doses <1.0 mg/kg. Additionally, the target ALP range was not achieved in many of these patients. Burosumab can be administered to children in doses up to 2.0 mg/kg (Kyowa Kirin Co., Ltd., 2023); thus, the dose of burosumab could still be increased if necessary.

This study had some limitations. Notably, there were some differences in the background characteristics between patients who were treated with burosumab and those who were not, which were not statistically adjusted for. Thus, the results of this analysis should be interpreted cautiously. Fractures were not evaluated owing to the small number of cases within 1 year. Insufficient data were available regarding the use of active vitamin D or oral phosphate prior to baseline, which may have affected the degree of renal calcification and phosphate/ALP levels. Additionally, the number of patients at each timepoint varied, which may have contributed to a potential assessment bias. Moreover, although an approximate time for collecting blood samples after drug administration was defined, the actual interval between drug administration and blood sampling may have been inconsistent. Finally, this analysis reports data from up to 1 year of burosumab treatment; therefore, any further changes to outcomes beyond 1 year of treatment are not yet known. Studies with a longer follow-up will be necessary to determine whether any long-term changes in RSS, height, or QoL correlate with ALP.

In conclusion, this study demonstrated an improvement in biochemical parameters following burosumab treatment for XLH in both children and adults. Additionally, there was a correlation between ALP and rickets severity in Japanese children with XLH, which may suggest that ALP could be used as a marker for monitoring XLH severity and improvement of XLH symptoms in response to treatment. These results also provide evidence for the importance of ALP monitoring during the management of XLH. As this study was an interim analysis, further insights are expected in future analyses with a longer follow-up.

## CRediT authorship contribution statement

**Keiichi Ozono:** Writing – review & editing, Writing – original draft, Methodology, Investigation, Conceptualization. **Hee Gyung Kang:** Writing – review & editing, Investigation. **Noriyuki Namba:** Writing – review & editing, Investigation. **Nobuaki Ito:** Writing – review & editing, Investigation. **Takuo Kubota:** Writing – review & editing, Investigation. **Ayumi Shintani:** Writing – review & editing, Methodology, Formal analysis, Data curation. **Ryota Kawai:** Writing – review & editing, Methodology, Formal analysis, Data curation. **Osamu Miyazaki:** Writing – review & editing, Investigation. **Masanori Kanematsu:** Writing – review & editing, Project administration. **Haruka Ishii:** Writing – review & editing, Methodology. **Toshimi Michigami:** Writing – review & editing, Investigation.

## Funding

This study was funded by Kyowa Kirin Co., Ltd.

## Declaration of competing interest

**Keiichi Ozono** has received consulting fees from Kyowa Kirin Co., Ltd.; payment from Kyowa Kirin Co., Ltd. and Alexion Pharmaceuticals, Inc.; and is a member of The Japanese Society for Pediatric Endocrinology. **Hee Gyung Kang** has received grants from Chong Kun Dang Pharmaceutical Corp., Handok Inc., Kyowa Kirin Co., Ltd., Amgen Inc., Apellis Pharmaceuticals, Inc., and AstraZeneca K.K.; consulting fees from Handok Inc. and Kyowa Kirin Co., Ltd.; payment from Alexion Pharmaceuticals, Inc., Handok Inc., and Kyowa Kirin Co., Ltd.; and participated on Data Safety Monitoring Boards or Advisory Boards of Bayer Yakuhin, Ltd. **Noriyuki Namba** has received consulting fees and payment from Kyowa Kirin Co., Ltd.; and is a member of The Japanese Society for Pediatric Endocrinology, The Asia Pacific Pediatric Endocrine Society, and The International Meeting of Pediatric Endocrinology. **Nobuaki Ito** has received research funding and consulting fees from Kyowa Kirin Co., Ltd.; the Division of Therapeutic Development for Intractable Bone Diseases, Graduate School of Medicine and Faculty of Medicine, The University of Tokyo is an endowment department supported by Kyowa Kirin Co., Ltd. **Takuo Kubota** has received research funding, consulting fees and payment from Kyowa Kirin Co., Ltd. **Ayumi Shintani** has received consulting fees from Chugai Pharmaceutical Co., Ltd., Daiichi Sankyo Co., Ltd., Kyowa Kirin Co., Ltd., Takeda Pharmaceutical Co., Ltd., and Shionogi & Co., Ltd.; and payment from Asahi Kasei Pharma Corporation, AstraZeneca K.K., Astellas Pharma Inc., Bayer Yakuhin, Ltd., Chugai Pharmaceutical Co., Ltd., Daiichi Sankyo Co., Ltd., Janssen Pharmaceutical K.K., Kissei Pharmaceutical Co., Ltd., Kyowa Kirin Co., Ltd., Mallinckrodt Pharma K.K., Merck Biopharma Co., Ltd., MSD K.K., Nippon Shinyaku Co., Ltd., Pfizer Japan Inc., and Takeda Pharmaceutical Co., Ltd. **Masanori Kanematsu** and **Haruka Ishii** are employees of Kyowa Kirin Co., Ltd. **Toshimi Michigami** has received research funding from Kyowa Kirin Co., Ltd.; consulting fees from Kyowa Kirin Co., Ltd. and Alexion Pharmaceuticals Inc.; and payment from Kyowa Kirin Co., Ltd., Alexion Pharmaceuticals Inc., Amgen Inc., Chugai Pharmaceuticals Co., Ltd., Novo Nordisk Pharma Ltd., and BioMarin Pharmaceuticals Inc. **Ryota Kawai** and **Osamu Miyazaki** do not have any conflicts of interest to declare.

## Data Availability

The data that has been used is confidential.
