# Supplementary material for: Burosumab improves alkaline phosphatase and rickets severity in X-linked hypophosphatemia: 3-year SUNFLOWER analysis
Source: Bone Rep. 2026 Aug 30;30:101944. doi: 10.1016/j.bonr.2026.101944 (PMC13584130; doi:10.1016/j.bonr.2026.101944)
Supplement: Supplementary file 1 [file mmc1.docx]

**Supplementary Materials**

**Burosumab improves alkaline phosphatase and rickets severity in X-linked hypophosphatemia: 3-year SUNFLOWER analysis**

Keiichi Ozono, Hee Gyung Kang, Noriyuki Namba, Nobuaki Ito, Takuo Kubota, Ayumi Shintani, Ryota Kawai, Osamu Miyazaki, Masanori Kanematsu, Haruka Ishii, and Toshimi Michigami

**Supplementary Figure S1.** Serum markers at baseline and up to 12 months in children (<18 years) who continued either burosumab or non-burosumab treatment for 1 year. (a) Phosphate, (b) serum calcium, (c) parathyroid hormone, (d) 1,25(OH)_2_D, and (e) ALP.

Horizontal lines indicate medians, box edges represent interquartile ranges, whiskers show the range of the remaining data within 1.5 times the IQR from the box edges, and data points outside the whisker range are displayed as dots. 1,25(OH)_2_D, 1,25-dihydroxyvitamin D; ALP, alkaline phosphatase; IC, informed consent; iPTH, intact parathyroid hormone; M, months.

**Supplementary Figure S2.** Serum markers at baseline and up to 12 months in adults (≥18 years) who continued either burosumab or non-burosumab treatment for 1 year. (a) phosphate, (b) serum calcium, (c) parathyroid hormone, (d) 1,25(OH)_2_D, and (e) BALP.

Horizontal lines indicate medians, box edges represent interquartile ranges, whiskers show the range of the remaining data within 1.5 times the IQR from the box edges, and data points outside the whisker range are displayed as dots. 1,25(OH)_2_D, 1,25-dihydroxyvitamin D, BALP, bone-specific alkaline phosphatase; IC, informed consent; iPTH, intact parathyroid hormone; M, months.

**Supplementary Figure S3.** Change in height Z-scores over time in children (<18 years) who continued either burosumab or non-burosumab treatment for 1 year.

(a) Patients not treated with burosumab. Horizontal lines indicate medians, box edges represent interquartile ranges, and whiskers show ranges. (b) Patients treated with burosumab. Data are individual datapoints, with the horizontal line indicating mean values and shaded gray areas representing standard deviations. BL, baseline; IC, informed consent; IQR, interquartile range; M, months.

**Supplementary Figure S4.** Renal calcification grade changes in (a) children (<18 years) and (b) adults (≥18 years) who continued either burosumab or non-burosumab treatment for 1 year. BL, baseline; IC, informed consent; M, months.

**Supplementary Table S1.** Target range for phosphate and ALP levels by age range

| **Phosphate, mg/dL** | **Male, LLN** | **Female, LLN** |
| --- | --- | --- |
| 1–4 years | ≥4.3 | ≥4.3 |
| 5–12 years | ≥4.1 | ≥4.1 |
| 13–15 years | ≥3.5 | ≥3.2 |
| 16–17 years | ≥2.9 | ≥2.9 |
| Adult (≥18 years) | ≥2.5 | ≥2.5 |
| **ALP, U/L** | **Male, ULN** | **Female, ULN** |
| 1–9 years | ≤369 | ≤369 |
| 10–12 years | ≤460 | ≤460 |
| 13–14 years | ≤517 | ≤280 |
| 15–16 years | ≤365 | ≤128 |
| 17 years | ≤164 | ≤95 |
| Adult (≥18 years)^a^ | 20.9 µg/L | 14.5 µg/L |

^a^ Bone-specific ALP

ALP, alkaline phosphatase; LLN, lower limit of normal; ULN, upper limit of normal.
